# Using giant african pouched rats to detect human tuberculosis: a review

**DOI:** 10.11604/pamj.2015.21.333.2977

**Published:** 2015-08-31

**Authors:** Alan Poling, Amanda Mahoney, Negussie Beyene, Georgies Mgode, Bart Weetjens, Christophe Cox, Amy Durgin

**Affiliations:** 1 Department of Psychology, Western Michigan University, Kalamazoo, Michigan 49008, United States of America; 2 APOPO, Tanzania; 3 Sokoine University of Agriculture, Tanzania

**Keywords:** Tuberculosis detection, pouched rats, mycobacterium tuberculosis, scent detection, discrimination learning, operant conditioning

## Abstract

Despite its characteristically low sensitivity, sputum smear microscopy remains the standard for diagnosing tuberculosis (TB) in resource-poor countries. In an attempt to develop an alternative or adjunct to microscopy, researchers have recently examined the ability of pouched rats to detect TB-positive human sputum samples and the microbiological variables that affect their detection. Ten published studies, reviewed herein, suggest that the rats are able to detect the specific odor of *Mycobacterium tuberculosis*, which causes TB, and can substantially increase new-case detections when used for second-line TB screening following microscopy. Further research is needed to ascertain the rats’ ability to detect TB in children and in HIV-positive patients, to detect TB when used for first-line screening, and to be useful in broad-scale applications where cost-effectiveness is a major consideration.

## Introduction

Tuberculosis is an infectious disease caused by *Mycobacterium tuberculosis*. About one-third of Earth's population has latent TB, which means that they have been exposed to *M. tuberculosis* but not become ill. About 10% of people with latent TB develop active TB, that is, they become ill with the disease, which in 2011 affected approximately 8.7 million people and killed 1.4 million of them [1]. Most deaths due to TB occur in resource-poor countries located in the tropics. Although TB is a treatable and curable disease, it causes immense suffering and many deaths in Africa, due in part to the absence of a cheap and reliable technique for detecting it. Sputum smear microscopy is the standard technique for diagnosing TB in resource-poor countries, due to its relatively low cost [2]. In this technique, sputum smears are stained with the Ziehl-Neelsen method, which causes *M. tuberculosis* bacteria to appear as bright red rods when viewed under a light microscope. Unfortunately, this technique misses many cases of TB. Although the technique's specificity (ability to detect the absence of active TB) is typically above 90%, which is good, the sensitivity of the method (ability to detect the presence of active TB) varies widely, ranging from about 20-80%, and is often poor [3]. In addition to suffering the consequences of untreated disease, each patient missed by microscopy (or any other diagnostic), and thus not treated, is likely to spread the disease to between 10 and 15 other people each year [1].

Alternatives to Ziehl-Neelsen microscopy, including fluorescent microscopy [3] and the GeneXpert assay [4], have been developed and are being adopted, but presently are not widely available in resource-poor areas [5]. A decade ago, Bart Weetjens, the founder of Anti-Persoonsmijnen Ontmijnende Product Ontwikkeling (APOPO; in English, Anti-Personnel Landmines Detection Product Development), was aware of the need to develop a viable alternative to or adjunct for microscopy for use in resource-poor areas. APOPO is a Belgian nongovernmental organization (NGO) headquartered in Morogoro, Tanzania that is widely recognized for its use of giant African pouched rats (*Cricetomys gambianus*) to detect landmines (e.g. [6, 7]). Weetjens knew of historical reports by physicians indicating that the breath and sputum of patients with consumption (as TB was once termed) often had a tarry odor, and he reasoned that this odor could be easily detected by pouched rats, which have a much better sense of smell than humans. Weetjens also hypothesized that laboratory procedures proven successful in teaching pouched rats to detect the odor of 2,4,6-trinitrotoluene (TNT), the primary explosive in most landmines, could be readily adapted to teach them to distinguish TB-positive from TB-negative samples, and he and his colleagues set out to explore the possibility of using pouched rats to detect human TB by sniffing human sputum. The purpose of this article is to review their findings to date.

## Methods

### Evidence that Pouched Rats can Detect TB

Pouched rats are large (25-45 cm body length, 1-2 kg body weight) burrowing rodents native to sub-Saharan Africa. Details of how the rats are maintained at APOPO are provided elsewhere [8]. In the first published report of their ability to detect active TB, Weetjens et al. [9] described a procedure in which the rats were trained in a rectilinear cage to sniff each of 10 holes located along the centerline of the cage's floor. A small pot containing a sputum sample taken from a patient at a Direct Observation of Treatment Short Course (DOTS) TB center in Dar es Salaam, Tanzania was placed immediately below each hole. In Tanzania, Zeehl-Nielson microscopy is the usual TB diagnostic at DOTS centers, although fluorescent microscopy and other techniques are occasionally employed. Weetjens et al. arranged for DOTS-center technicians to evaluate each sample via microscopy and freeze the remaining material, which was transported to APOPO's lab for analysis by the rats. In this and other studies, each patient typically provided two sputum samples, which is standard practice at the DOTS centers. Prior to use in training, each sputum sample was thawed and heated to inactivate *M. tuberculosis* and other infectious micro-organisms. The rats were trained by operant conditioning to pause for at least 5 seconds at holes where the sputum sample was positive for TB (as confirmed by microscopy) but not to pause at holes where the sputum sample was TB-negative. In essence, the training procedure involved differential reinforcement, arranged so that rats (which were mildly food deprived) received food only when they paused for the required time (5 s) at holes above TB-positive samples. When the discrimination was being established, the trainers were aware of which samples were TB-positive and TB-negative, so that they could arrange reinforcement (rewards) appropriately. In testing the system, however, trainers did not know the status of several samples (termed “unknown”, and rats’ performance on unknown samples was used to index their sensitivity and specificity in detecting TB.

In the Weetjens et al. [9] study, two rats were tested with unknown samples that were confirmed as TB-positive (67 samples) or TB-negative (750 samples) by culturing, which is the “gold standard” for TB detection. The sensitivity of each individual rat was 73.1%. That is, each rat identified as TB-positive 49 of the 67 sputum samples identified as TB-positive by culturing. The specificity of one rat was 93% and that of the other rat was 93.8%. That is, the first rat identified as TB-negative 698 of the 750 sputum samples identified as TB-negative by culturing and the second rat identified 703 of these samples as TB-negative. Weetjens et al. [9] also compared rats’ evaluation of sputum samples to evaluations made by trained DOTS-center microscopists. Summary results were presented for 16 rats that evaluated 2,597 sputum samples, of which 345 were smear-positive. The mean sensitivity of these rats was 87.9% and the mean specificity was 93.3%. More detailed analysis of the performance of three animals yielded comparable results.

## Current status of knowledge

These findings were sufficiently promising that the Tanzanian Ministry of Health allowed the rats to be used for simulated second-line screening of sputum samples provided by DOTS-center patients. In a study by Weetjens et al. [10], samples from 15,041 patients were evaluated by microscopy at the DOTS centers and by the rats. The DOTS centers found TB in 1,838 of the patients (12.2%), whereas the rats detected the disease in 2,415 patients (16.1%). The cases detected by rats but missed by DOTS centers (n = 577) increased TB detection by 31.4%, which is statistically, and more importantly clinically, significant. In this study, a second microscopy was used to confirm the status of samples indicated by the rats as TB-positive. The results of screening sputum samples from additional DOTS-center patients were analyzed by Poling et al. [11], who reported that in 2009 23,101 sputum samples from 10,523 patients were evaluated, first by DOTS-microscopists and then by the rats. DOTS centers identified 2,487 TB-positive sputum samples and 1,335 TB-positive patients. The rats indicated 2,085 new samples and 898 new patients at TB-positive. Of these, 927 samples and 620 patients were confirmed by APOPO microscopy to be positive for TB. Thus, the rats increased the new case detection rate by 44%. Poling et al. also reported individual sample-wise data for 10 rats. The average sensitivity was 86% (range 82-89%) and the average specificity was 93% (range 91-95%). As in other studies, Poling et al. notified the appropriate DOTS center whenever a DOTS-negative patient was deemed TB-positive by APOPO, and the DOTS center arranged appropriate activities regarding that patient (i.e., contact and treatment). Mahoney et al. [12] reported that in 2010 *Crycetomys* evaluated sputum samples from 12,347 patients, 1,671 (13.5%) of whom were identified as TB-positive by DOTS center microscopy. The rats identified an additional 716 patients as TB-positive, a 42.7% increase in the TB detection rate. Again, individual data were calculated for 10 rats and average sensitivity was 72% (range 69-77%) and average specificity was 86% (range 82-89%). With the exception of two rats that evaluated 67 sputum samples confirmed by culturing to be TB-positive and 750 samples confirmed to be TB-negative [9], the sensitivity and specificity of pouched rats in detecting TB has been confirmed by a second microscopy. This is not a strong confirmatory technique and was dictated solely by the unavailability of a better alternative. To examine the rats’ performance relative to the “gold standard” for TB detection, Mahoney et al. [13] had rats evaluate 910 sputum samples previously evaluated by smear microscopy. All samples were also evaluated through culturing and multiplex polymerase chain reaction (PCR) was performed on culture growths to classify the bacteria. The patient-wise sensitivity of microscopy was 58.0%, and the specificity was 97.3%. Used as a group of 10 with a cutoff (defined as the number of rat indications to classify a sample as positive for *M. tuberculosis*) of 1, the rats increased new case detection by 46.8% relative to microscopy alone. The average sensitivity of the individual rats was 68.4% (range 61.1-73.8%), and the mean specificity was 87.3% (range 84.7-90.3%). These results, coupled with those of prior studies using a less robust confirmatory technique, suggest that pouched rats are a valuable adjunct to, and may be a viable substitute for, sputum smear microscopy as a TB diagnostic in resource-poor countries. To date, the rats have detected over 3,000 cases of TB missed by microscopy.

### Improving operational effectiveness and efficiency

Pouched rats can evaluate sputum samples very quickly, but considerable time is required to prepare samples for evaluation by the rats. Recent studies have evaluated possible strategies for reducing the time required to process samples. In one study, Mahoney et al. [14] explored whether pouched rats could accurately detect active TB by sniffing fixed microscope slides. Because such slides are prepared for each sample at DOTS centers and would require no further processing before being presented to the rats, their use would substantially speed sample evaluation by the rats. In the Mahoney et al. [14] study, six trained pouched rats evaluated 35 DOTS-positive sputum and 315 DOTS-negative sputum samples and six other rats evaluated fully prepared smears (slides) from those same samples. All of the DOTS-positive samples contained high concentrations of *M. tuberculosis*, which was intended to make them easy for the rats to detect. Because the rats can evaluate samples quickly, in operational use it is possible to have each sample evaluated by more than one animal. When this is done, a decision must be made with respect to the proportion of animals identifying a sample as TB-positive for the sample to be so considered. That is, a specific decision criterion must be determined. Mahoney et al. reported sensitivity and specificity data using all possible decision criteria (i.e., 1, 2, 3, 4, 5, and 6 of 6 positive). In this study, accuracy in evaluating fully prepared smears (microscope slides) was highest at a cutoff of 4 of 6 positive indications. With this criterion, when samples were presented as microscope slides, sensitivity was 70.5% and specificity was 81.7%. The optimal cutoff in the sputum condition was 3, which yielded a sensitivity of 94.4% and a specificity of 94.4%. These results suggest that the rats can detect TB in prepared slides, but that their accuracy in doing so is substantially lower than their accuracy in detecting TB in sputum. Although further research investigating TB detection from fixed slides is warranted, in order to maximize new case detection APOPO presently evaluates only sputum presented in pots. Early-on in the process of developing the TB diagnostic, a decision was made to add 5 ml of phosphate-buffered saline solution (PBS) to each sputum sample prior to evaluation by the rats. The rationale for doing this was that adding the liquid should increase the volatiles available for detection by the rats. No confirmatory data were collected, however. A time analysis conducted across two consecutive sample-processing days indicated that the use of PBS added 70.2 minutes to the time required to process 600 samples [15], which is warranted only if addition of the solution improves the rats’ performance. To determine whether it does so, Mahoney et al. (15, Experiment 1) arranged a study in which 10 trained rats evaluated an equal number of sputum samples with and without PBS added. A within-subjects experimental design was used and the results revealed that there was no substantial difference in the sensitivity or specificity of individual rats, or of the 10 rats as a group, in evaluating samples with or without added PBS. The mean sensitivity of the rats was the same, 75.6%, when PBS was and was not added to samples. The mean specificity was 77.9% when PBS was added and 80.1% when PBS was not added. In view of these results, APOPO personnel no longer add PBS to samples.

By convention, microscopists count the number of bacteria they observe and categorize TB-positive samples according to bacterial load, from AFB (a few bacteria), through the progressively increasing categories of +1, +2, and +3 [16]. It is much harder for microscopists to detect low concentrations of *M. tuberculosis* than to detect high concentrations, and APOPO receives relatively few AFB and +1 samples. Training the rats with a preponderance of high-concentration samples may well reduce their accuracy in detecting low-concentration samples, and artificially inflating the number of low-concentration training samples they receive may improve their accuracy. Mahoney et al. (15, Experiment 2) evaluated this possibility by training five rats with samples as they were received from DOTS centers with no regards to bacteria load. They trained five other rats only with AFB and +1 samples. When all rats were subsequently tested with +1 samples, the mean sensitivity of the rats trained with all samples was 55% and the mean sensitivity of the rats trained with AFB and +1 samples was 82.8%. The difference in mean sensitivity was statistically significant. Mean specificities for these respective groups were 85.6% and 82.5; the difference in means was not statistically significant. These findings suggest that training on low concentration samples improves rats’ detection of such samples. Unfortunately, insufficient low-concentration sputum samples are available to make practical use of these findings.

### Microbiological variables that affect detection

Sputum from patients at DOTS centers contains microorganisms other than *M. tuberculosis*, some virulent, and it is possible that the presence of these microorganisms affects the rats’ performance. For example, *Mycobacteria* other than *M. tuberculosis* (non- *Mtb* species, such as *M. bovis, M. avium*) might be present and produce odors similar to those produced by *M. tuberculosis*. Further, nonmycobacterial species found in the respiratory tract may produce an odor similar to that of *M. tuberculosis*. If so, the rats would err by identifying microorganisms other than *M. tuberculosis* as TB-positive. Mgode and his colleagues investigated this issue in a series of experiments [17–19] designed to determine whether the rats falsely identify nonmycobacterial species and falsely identify non- *Mtb* mycobacterial species as *M. tuberculosis*, and which compounds within the M. tuberculosis species play an important role in detection by rats. In the first experiment in the series [17] sputum samples from 289 subjects were analyzed by smear microscopy, culture, PCR, and the rats. A group of 10 rats evaluated 514 processed sputum samples. The sputum samples were evaluated for the presence of *M. tuberculosis*, non-tuberculous mycobacteria (*Mycobacterium* species other than *M. tuberculosis*) (NTM), and six other microorganisms of the respiratory tract (*Nocardia* spp., *Rhodococcus* spp., *Streptomyces* spp., *Moraxella* spp, Candida spp., and *Streptococcus pneumonia*). All samples evaluated for the presence of *Mycobacteria* (n = 380) were cultured on Lowenstein Jensen media to isolate mycobacterial species and subjected to multiplex real-time PCR. Conventional multiplex PCR was additionally conducted. Nonmycobacterial respiratory tract microbes were also cultured from 394 sputum samples and isolates were identified by colony and cell morphology and biochemical tests. Those samples found negative for *Mtb* by smear microscopy and culture but found positive by the rats underwent gas chromatography/mass spectrometry (GC/MS) to identify volatile compounds of the microorganisms. Test results yielded 19 smear microscopy-positive sputum cases, 37 culture-positive cases, and 5 TB-suspect cases. The rats identified 45 of the 56 confirmed TB cases. The rats also identified 63 of the 228 TB-negative patients as TB-positive. Thus, at a cutoff of two rat agreements, patient-wise sensitivity was 80.4% while patient-wise specificity was 72.4%. About 40% of *NTM, M. avium* subsp., *hominissuis*, and *M. intracellulare* samples were detected by the rats, which is significantly lower than the detection rate on *Mtb* (p= .054, Fisher's exact test) but also lower than the rats’ detection accuracy on some nonmycobacterial species. Three nonmycobacterial species were identified that produced relatively high detection rates: *Rhodoccocus* (2 out of 3 samples), *Nocardia* (2 out of 3 samples), and *Streptomyces* (4 out of 10 samples). There were too few samples available in these categories to reach a conclusion regarding the rats’ detection accuracy on these samples and the odor analysis failed to identify *Mtb* markers common to these other microorganisms. Rat false alarm rates in smear-negative sputum containing other nonmycobacterial species ranged from 0-33.3%. In total, the rats indicated 28 of 111 smear-negative samples (25.2%) containing one or more of the tested respiratory tract microbes to be TB-positive.

In summary, the rats’ detection accuracy on samples containing *Mtb* was significantly higher (p < .05) than accuracy on other nonmycobacterial species except *Rhodococcus* spp., *Nocardia* spp., and *Streptomyces* spp. (p > .05), but the odor analysis results indicated that these species do not produce candidate odor markers produced by *Mtb*. It is possible that sputa found negative by smear microscopy and culture is in fact positive for TB but below the detection thresholds of these tests. It is also possible that the rats’ detection of these sputum samples is based upon odors unassociated with these microorganisms but instead are associated with necrotic materials or other byproducts found in the respiratory tract produced in response to illness. This possibility is reasonable given that all sputum samples were collected from TB clinics and thus all patients exhibited symptoms of a respiratory illness. It could potentially be evaluating by determining the rats’ evaluation of sputa from non-symptomatic people. In the second study of the series [18], diagnostic performance of the rats on pure cultures mixed into negative sputum was compared with performance on naturally TB-infected sputum. Pure cultures of *Mtb*, NTM, clinical *Mtb* isolates, Nocardia spp., Rhodococcus sp., Streptomyces spp., Bacillus sp., Candida sp., and Saccharomyces sp. were tested. Freeze-dried bacterial strains were reconstituted and inoculated into 14-20 ml of Middlebrook liquid medium, checked for purity, and incubated. All test organisms were heat inactivated in a 90° C water bath for 30 min and stored at -20°C until used. Negative sputum samples, collected from TB clinics, were confirmed negative by smear microscopy (ZN and FM), mycobacterial culture, and the rats. These samples were exposed to the same inactivation and storage procedures as the test microorganisms. Negative samples were spiked with the culture isolates and presented to the rats across a period of 94 days. Further, 36 cultures were presented at various growth stages. Different volatiles may be associated with the various phases and this test provided information regarding the most detectable phase of growth. Each session, six rats evaluated samples containing 9 test microorganisms, 7 TB-positive controls used as reinforcement samples for the rats (these samples were mixed with a sterile medium), and 54 negative controls (also mixed with a sterile medium). Unlike the prior study, this study found that the rats did not detect mycobacterial-related *Nocardia* spp., *Rhodococcus* sp., or other microbes. The rats discriminated *Mtb* from other microbes (p <.008) and sensitivity on naturally TB-positive sputum was higher than sensitivity on sputum spiked with *Mtb* cultures (50%). The analysis of rat accuracy on various stages of culture indicated that the stage of growth affects performance. Very young cultures (≤ 10 days) or old cultures (≥ 41 days) yielded much lower sensitivity than cultures taken between 21 and 30 days of growth; overall sensitivity was 50% while sensitivity on 11-30-day cultures was 83.3%. Specificity was 94.4% on negative control samples. This study indicated that the rats perform better on naturally-infected TB positive sputum and that different stages of culture growth yield different levels of sensitivity. That the rats perform better on naturally-infected TB- positive sputum suggests that background odors differed in these samples and implies that characteristics of the host body may affect the rats’ performance. Therefore, evaluation of the performance of rats in several different populations, such as patients at DOTS centers in dissimilar cities and inmates in rural prisons, would be useful to determine whether detection accuracy varies due to background odors. The results of these two studies provided information regarding the mycobacterial species and other microorganisms that the rats detect in addition to *Mtb*. In a third study [19], specific odor compounds characteristic of *Mtb* were tested. The purpose of the study was to determine the volatile compounds of *Mtb*-positive sputum detected by the rats and whether other microorganisms related to TB, which also cause pulmonary disease, are detected by the rats on the basis of these odor compounds. The microorganisms studied were non-*Mtb*, *Nocardia* spp., *Streptomyce*s spp., clinical isolates,*Rhodococcus* sp., Staphylococcus sp., and Candida sp. To identify volatile compounds, headspace samples from the microorganisms in various media and growth-phases were collected via a closed-loop apparatus fitted with an activated charcoal filter for 18-24 h. Compounds recovered from at least two cultures, but absent from the blank media, were considered significant. Thirteen volatile compounds specific to *Mtb* and 13 shared with other microorganisms were found. Eight volatiles unique to *Mtb* and seven volatiles shared between *Mtb*, non-*Mtb* mycobacteria, and other microorganisms were presented to the rats. The compounds were presented first individually to the rats and later in pairs and blends.

Results indicated that rat detection of TB-negative sputa spiked with a blend of 6 *Mtb*-specific volatile compounds was significantly different (p=.001) from detection of TB-negative sputa spiked with shared volatile compounds. None of these compounds, when presented individually, were identified by the rats as TB-positive at a statistically significant level. Further, the blend of the three most abundant *Mtb*-specific compounds (methyl nicotinate, methyl 4-anisate and 2-phenylanisol) was not detected at a statistically significant level. Thus, this study indicates that TB detection by the rats involves a relatively complex odor profile that likely becomes even more complex when dealing with live human subjects. Further, aligned with other scent-detection research [20, 21], it suggests that odors detectable as a blend may not evoke the relevant detection response when presented singly.

## Conclusion

Although scientific interest in using animals to detect disease is growing, as evidenced by the recent publication of two reviews examining dogs’ ability to detect ill health in humans [22, 23], this general strategy is relatively new, rarely used, and not generally accepted by the medical community. Widespread application of any animal-based diagnostic, including the use of *Cricetomys* to detect TB, will depend upon the availability of compelling data showing that the diagnostic is acceptably accurate and financially viable. Although researchers have made considerable progress in evaluating pouched rats as second-line TB detectors, they have not yet produced an exportable TB-detection system that has been independently verified as useful on a broad scale. Moreover, the value of pouched rats as second-line TB screens depends on the quality of the first-line screen. Put simply, if microscopy were highly accurate, there would be no need for the rats. There is a strong international push to develop and make widely available a fast, inexpensive, and accurate alternative to smear microscopy and, when it arrives, there will be no need for second-line screening of any type. In the interim, which is of uncertain and potentially lengthy duration, the rats may be of clinical value and further examination of how best to use them for second-line screening appears to merit attention. So, too, does the possibility of using *Cricetomys* for first-line screening in under-served areas with high TB prevalence. Finally, further research is clearly needed to ascertain the rats’ ability to detect TB in HIV-positive patients and in children, which were not treated as independent groups in the studies conducted to date.
